# A case‐by‐case analysis of EPN and LPP components within a “one‐picture‐per‐emotion‐category” protocol

**DOI:** 10.1111/psyp.14718

**Published:** 2024-11-12

**Authors:** Harald T. Schupp, Karl‐Philipp Flösch, Tobias Flaisch

**Affiliations:** ^1^ Department of Psychology University of Konstanz Constance Germany; ^2^ Centre for the Advanced Study of Collective Behaviour University of Konstanz Constance Germany

**Keywords:** attention, case‐by‐case, emotion, EPN, ERPs, LPP

## Abstract

Stimuli encountered in the environment are continuously evaluated according to their affective stimulus significance. Numerous event‐related potential studies have shown that the early posterior negativity (EPN) and the late positive potential (LPP) are larger for high than low arousing emotional pictures. The group approach has been recently extended to the study of the individual case. Usually, many exemplars are used to represent an emotion category. Determining how many pictures are needed to reliably assess affective stimulus evaluation processes at the individual level is crucial when moving toward the goal of exploring idiosyncratic emotional stimuli. Accordingly, in the present study (*N* = 16), singular images displaying erotic, neutral, and mutilation content were shown 800 times while dense sensor EEG was recorded. At the group level, enhanced EPN and LPP amplitudes for high compared to low arousing stimuli emerged. At the single subject level, significantly larger amplitudes to the erotic than neutral image were observed in 15 out of 16 tests for the EPN and LPP components. Regarding the mutilation image, 15 participants showed a significant EPN effect, while the LPP effect was only found in 10 cases. Notably, emotional modulation of the EPN and LPP was stable over time. The present study contributes to the development of experimental designs tailored to the needs of the case‐by‐case approach. Since the process of affective stimulus evaluation is considered as a process common‐to‐all, the use of a singular stimulus exemplar may prove useful to investigate the idiosyncratic nature of emotion.

## Abstract 2

A case‐by‐case approach has recently demonstrated that emotional modulation effects of the early posterior negativity and late positive potential components can be assessed at the level of the individual case. Here, we explored a protocol in which picture categories are represented by a singular image rather than multiple exemplars. Findings show that emotion effects can reliably be observed for singular stimuli and encourage the pursuit of an individualized approach to study the process of affective stimulus evaluation.

## 1 INTRODUCTION

In a world where various stimuli compete for attentional resources, the quick and reliable detection of hospitable and hostile stimuli enhances adaptive behavior (Lang et al., 1997; Öhman et al., 2000). Functional and evolutionary considerations suggest the preferential processing of emotional cues. Event‐related brain potential (ERP) studies can reveal the unfolding of emotional stimulus processing within the first few hundred milliseconds (Schupp, Flaisch, et al., 2006). Two ERP components—early posterior negativity (EPN) and late positive potential (LPP)—are consistently modulated by the significance of emotional stimuli. The EPN component, observed as a relative negative difference over temporo‐occipital sites between 150 and 350 ms, is more pronounced when processing emotional (pleasant and unpleasant) pictures compared to neutral ones (e.g., Junghöfer et al., 2001; Schupp et al., 2003; Schupp, Flaisch, et al., 2006). The LPP component, measured over centro‐parietal regions between 300 and 700 ms, is larger for emotional stimuli than for neutral stimuli (e.g., Cuthbert et al., 2000; Sabatinelli et al., 2013). Furthermore, the emotional modulation of the EPN and LPP is most accentuated for pictures depicting evolutionarily significant stimuli, such as scenes related to reproduction and defense (Schupp et al., 2003; Schupp, Flaisch, et al., 2006), which also provoke reliable somatic, autonomic, and humoral responses (Bradley et al., 2001). These findings have been considered from the perspective of “natural selective attention,” which holds that emotionally arousing—pleasant and unpleasant—pictures guide selective visual attention processes, thereby serving as indicators of affective stimulus significance (Bradley, 2009; Lang et al., 1997; Schupp, Flaisch, et al., 2006).

Until recently, emotional modulation effects of EPN and LPP components were based on group analysis (Hajcak et al., 2010; Schindler & Bublatzky, 2020; Schupp, Flaisch, et al., 2006). A complementary approach to reveal principles of emotion processing is to study the effect at the individual level. This case‐by‐case approach can reveal principles of emotion processing common to all individuals in the research sample, rather than reflecting the group as an aggregate whole, that is, a “hypothetical person” (Danziger, 1990; Lamiell, 1998; Robinson, 2011). A series of studies used this approach to assess emotional stimulus processing at the individual level (Schupp et al., 2023; Schupp & Kirmse, 2021, 2022). Pooling the findings across the three studies, the majority of participants showed larger EPN and LPP components to high‐ compared with low‐arousing stimuli for pictures displaying erotica (EPN: 100%, LPP: 100%, out of 51 tests), mutilations (EPN: 92%, LPP: 98%, out of 52 tests), and threatening animals (EPN: 94%, LPP: 90%, out of 50 tests). Intersubject replication of the effects within and across these studies (direct and systematic replication; Sidman, 1960) provided evidence that these two ERP components can serve as indicators of affective stimulus evaluation at the individual level.

Assessing affective stimulus evaluation at the individual level is relevant for basic research and the development of biomarkers, facilitating the translation of fundamental research to clinical applications. However, there is a conundrum: on the one hand, emotions—and what people find emotional—vary greatly from person to person; on the other hand, there is a search for universal laws of emotion that reflect common empirical principles (Frijda, 1988). One may reason that it is the meaning and significance attributed to external stimuli encountered in the environment that vary from person to person, rather than the process by which emotionally significant stimuli capture attention (Frijda, 1988; Lazarus, 1991). Taking a first step in this direction, the experimental protocol was refined regarding the number of stimuli representing an emotion category of interest. Previous research used many instances to represent a single category, with studies ranging between five and several hundred images per category (Flaisch, Junghöfer, et al., 2008; Hajcak et al., 2010; Junghöfer et al., 2001; Schupp et al., 2004). This approach can minimize differences in low‐level physical stimulus characteristics between the emotion categories (e.g., Delplanque et al., 2007; Junghöfer et al., 2001). Furthermore, it can reveal the relationship between the spatial frequencies of images and emotion processing (De Cesarei & Codispoti, 2013), as well as identify emotionally diagnostic sensory‐perceptual features (Farkas et al., 2020). However, empirically assessing idiosyncrasies seems feasible only when emotion categories can be represented by a few—ideally just one—stimulus exemplars. For instance, presenting individually tailored stimuli alongside standardized images from behavioral systems of sexual reproduction and disease avoidance could help probe individual differences in affective relevance.

A “one‐picture‐per‐emotion‐category” protocol can also provide new insights into affective habituation processes. Until now, experimental findings have not shown that habituation has a strong effect on the preferential processing of affective stimuli as reflected by the EPN and LPP. For instance, one study repeated 10 pleasant, 10 unpleasant, and 20 neutral stimuli 90 times, resulting in a total of 3600 picture presentations. Across 18 blocks of repetition, little evidence for habituation was found, and the emotional modulation of the EPN was similar in the last block compared to the first (Schupp, Stockburger, et al., 2006). A somewhat more nuanced picture is seen for the LPP. On one hand, Codispoti et al. (2016) found sustained emotional LPP modulation over time by repeating five pleasant, five neutral, and five unpleasant stimuli in three habituation blocks involving 21 stimulus repetitions. On the other hand, other studies reported affective habituation of the LPP, indicating a decrease in emotional modulation with stimulus repetition (Ferrari et al., 2020, 2022; Mastria et al., 2017). However, although the magnitude diminished, emotional modulation effects remained significant over time in all three studies and were even observed when the stimulus materials (two pleasant, two neutral, and two unpleasant images) were presented in two separate sessions, each consisting of 80 repetitions (Ferrari et al., 2020). A one‐picture‐per‐emotion‐category protocol with massive stimulus repetitions expands previous research in two respects. First, at the group level, using one picture per category further reduces the novelty of the stimulus materials, potentially revealing affective habituation in a highly predictable context. Second, case‐by‐case analyses tap into the assessment of interindividual differences in affective habituation.

The main aims of the present study were to assess the effects of emotional modulation and affective habituation on the EPN and LPP components using a single picture to represent each emotion category. To this end, one erotic image, one image depicting mutilation, and one control stimulus featuring a neutral person were repeatedly presented, resulting in 800 presentations of each image. First, group analyses were conducted to determine whether emotional modulation of EPN and LPP components could be observed within this one‐picture‐per‐emotion‐category protocol. Second, we tested whether these effects could be detected at the individual level and determined the proportion of cases showing increased EPN and LPP components in response to highly emotionally arousing pictures compared to low arousing ones. Third, we explored whether these effects are subject to affective habituation at both the group and individual levels, specifically examining whether a decrease in emotional modulation of the EPN and LPP would be observed across stimulus repetitions.

## 2 METHODS

### 2.1 Participants

Sixteen students (8 female; 15 right‐handed, 1 ambidextrous) between 20 and 33 years of age (*M* = 23.6) participated in the study. All possessed normal or corrected‐to‐normal vision and reported no neurological or psychiatric illnesses. Participants were recruited at the University of Konstanz and received course credit or monetary compensation. The study was approved by the ethics committee of the University of Konstanz in accordance with the declaration of Helsinki. Before the experiment, all participants were informed about the procedure. After they had been familiarized with exemplary pictures representative of the experimental picture set, written informed consent was obtained from all participants.

### 2.2 Stimuli

Pictures were selected from the International Affective Picture Series (IAPS; Lang et al., 2008). For the main experiment, the picture set comprised six colored pictures, which were subdivided into two subsets, each containing one picture displaying a heterosexual couple in an erotic setting (IAPS code: 4690, 4611), one picture of a single person in a neutral every‐day setting (2383, 2372) and one image of a mutilated body (3130, 3010). The images were overlaid with a white fixation cross in a black box (22 × 22 pixels) which was positioned in the center of the image. For collecting self‐report data, 25 additional IAPS images were added,^1^ comprising scenes of animals (happy, angry, neutral) and people (romantic, neutral, threatened). One mutilation picture of the experimental set (3010) was inadvertently replaced in the rating experiment and thus no rating data was acquired for this image.

To confirm a priori selection criteria for the experimental stimulus set, participants rated the stimulus materials for valence and arousal before and after picture presentations. Two‐factorial ANOVAs with repeated measurements on the factors Picture Category (erotic vs. neutral vs. mutilation) and Time (pre vs. post) confirmed that the emotional images were rated higher in arousal than the neutral ones (Picture Category: *F*(2, 30) = 14.9, *p* < .001, $\eta_{p}^{2}$ = .498). Holm‐corrected post hoc comparisons indicated that this was the case for erotic (*t*(15) = 5.97, *p* < .001; *M*_erotic_ = 4.8, SD_erotic_ = 1.6; *M*_neutral_ = 2.7, SD_neutral_ = 1.3), as well as mutilation images (*t*(15) = 4.53, *p* < .001; *M* = 5.4, SD = 2.1), which did not differ from each other (*t*(15) = −1.16, *p* = .265). Interestingly, these findings were unaffected by whether the rating occurred before or after the experimental presentation (Time: *F*(1, 15) = 0.67, *p* = .427, $\eta_{p}^{2}$ = .043; Time by Picture Category: *F*(2, 30) = 2.66, *p* = .086, $\eta_{p}^{2}$ = .151). A similar picture emerged when analyzing the valence ratings. There was a highly significant main effect of Picture Category (*F*(2, 30) = 99.0, *p* < .001, $\eta_{p}^{2}$ = .868) which was also accompanied by non‐significant effects for Time (*F*(1, 15) = 3.38, *p* = .086, $\eta_{p}^{2}$ = .184) and Time by Picture Category (*F*(2, 30) = 0.34, *p* = .712, $\eta_{p}^{2}$ = .022). Here, erotic images (*M* = 6.9, SD = 0.8) were rated as more pleasant than neutral ones (*t*(15) = 8.36, *p* < .001; *M* = 4.7, SD = 0.6), which in turn were more pleasant than the mutilation pictures (*t*(15) = 6.53, *p* < .001; *M* = 2.2, SD = 1.2).

### 2.3 Procedure

At the beginning of the session, participants were asked to rate the pictures of both experimental subsets as well as the additional picture set according to their valence and arousal using the SAM rating scale (Bradley & Lang, 1994). Then the participants passively viewed the experimental sequence which consisted of 2700 picture presentations. First, one subset of three images was repeated 800 times (i.e., 2400 trials), then the complementary subset was repeated 100 times (i.e., 300 trials). Afterwards, participants used the SAM scale to rate the pictures again on valence and arousal dimensions.

For the electroencephalogram (EEG) condition, the order of subsets was balanced across participants. To generate a unique pseudo‐randomized trial sequence for each individual, the algorithm allowed a maximum of four repetitions of the same image and ensured that stimulus transitions between consecutive trials (i.e., the emotion categories presented in trials N‐1 and N) approximated equal frequencies across the 9 possible combinations. Similar to prior research involving a high number of picture presentations (e.g., Schupp et al., 2023; Schupp & Kirmse, 2021, 2022), three breaks were incorporated at approximately 10‐minute intervals, allowing participants to adjust their posture and rest. The sequence of images was pseudo‐randomized with the constraint that maximally four repetitions of the same image were allowed and that the frequencies of each possible transition were approximated. Each participant viewed a different sequence of images. In each trial, one stimulus was presented for 118 ms, which was immediately followed by a blank screen shown for 882 ms.

Using Presentation software (Neurobehavioral Systems, Inc., Albany, CA), the pictures were shown on a 20‐inch CRT‐monitor (75 Hz refresh rate, resolution 1024 × 768) located approximately 100 cm in front of the participant. At a picture resolution of 640 × 480 pixels, the images subtended approximately 14° horizontally and 11° vertically. Participants were instructed to keep their eyes comfortably focused on the center of the screen, and to simply view the pictures.

### 2.4 EEG recording and preprocessing

Brain and ocular scalp potentials were measured with a 256‐lead geodesic sensor net (GSN 200 v2.0; EGI: Electrical Geodesics, Inc., Eugene, OR), on‐line bandpass filtered from 0.01–100 Hz, and sampled at 250 Hz using Netstation acquisition software and EGI amplifiers. Electrode impedance was kept between 30 and 70 kΩ, as recommended by EGI guidelines for this type of EEG amplifier. Data were recorded continuously using the vertex sensor as a reference electrode. Using EMEGS software (Peyk et al., 2011), the continuous EEG data were offline filtered using a digital low‐pass filter with a half‐power cutoff at 40 Hz (Butterworth IIR filter, order 19, stopband: −45 dB at 50 Hz).

The data were then segmented and corrected for ocular artifacts based on a multiple regression method (Miller et al., 1988). Artifact rejection was then performed based on an elaborate method for statistical control of artifacts, specifically tailored for the analysis of dense sensor EEG recordings (Junghöfer et al., 2000). Afterwards, the data were re‐referenced to an average reference and baseline‐adjusted (100 ms pre‐stimulus). Finally, average waveforms were calculated separately for the three picture categories. Trial numbers for erotica (*M* = 664.9, SD = 39.1), neutral people (*M* = 654.3, SD = 42.9) and mutilations (*M* = 661.2, SD = 47.0) did not differ across conditions, *χ* ^2^(2) = 0.094, *p* = .954. To maintain equal representation of both emotional and neutral trials in the bootstrap analyses, trial counts were balanced post‐artifact rejection across conditions. This was achieved by aligning the trial counts between conditions, with the condition containing more trials matched to the number of trials in the condition with fewer trials through random selection.

### 2.5 Data analyses

#### 2.5.1 Group analyses: Emotion effects

Average waveforms were calculated separately for the erotic, mutilation, and neutral image including the first 2400 trials in which the same set of three images was repeated 800 times^2^. Findings from visual inspection and single sensor waveform analysis were used in concert to select bilaterally symmetrical sensor clusters and time windows to score the EPN and LPP amplitudes used in the group analysis.

For the EPN, a posterior negativity emerged for both, erotica and mutilation pictures compared to neutral images with a similar topography and timing. Accordingly, the EPN was scored in an occipito‐parietal sensor cluster comprising the following sensors: 81, 82 (TP9), 83, 84 (P7), 90, 91, 92, 93, 94, 95, 96 (PO7), 101, 102, 103, 104, 105, 106 (P9), 107, 112, 113, 114, 115, 116, 117, 121, 122, 123, 124, 125, 126, 134, 135, 136, 137, 138, 139, 146, 147, 148, 149, 150, 151, 157, 158, 159, 160, 161, 162 (PO8), 166, 167, 168, 169, 170 (P10), 171, 172 (P8), 175, 176, 177, 178, 179, 180, 188, 189, 190, 191, 192 (TP10), 200, 201, 202, 209, 210, 217, 218, 219, 232, and 256.

For the LPP, pronounced differences were observed regarding the topography of the emotion effect for the erotic and mutilation picture. Accordingly, the LPP was scored in different clusters. The contrast of mutilation with the neutral images was based on a centro‐parietal cluster comprising the following sensors: 59 (CP3), 65, 76, 77, 78 (CP1), 79, 84 (P7), 85 (P5), 86 (P3), 87 (P1), 88, 89, 97, 98 (PO3), 99, 100 (Pz), 109 (O1), 110, 111 (POz), 120, 129, 130, 131, 132, 141 (O2), 142 (PO4), 143 (P2), 144 (CP2), 153, 154 (P4), 155, 156 (CP4), 163 (P6), 164, 165, and 172 (P8). The comparison of erotica with the control pictures was conducted on a cluster shifted anteriorly: 7, 8 (FCz), 9, 16, 17, 24 (FC1), 41 (FC3), 42, 43 (C1), 44, 49, 50, 51, 52, 57, 58 (C3), 59 (CP3), 64, 65, 70, 71, 77, 78 (CP1), 79, 80, 87 (P1), 88, 89, 99, 100 (Pz), 111 (POz), 130, 131, 132, 133, 143 (P2), 144 (CP2), 145, 155, 156, 165, 174 (CP6), 182, 183 (C6), 184 (C4), 185, 186 (C2), 187, 196, 197, 198, 199, 206, 207 (FC4), 208 (FC2), and 257 (Cz).

Regarding the timing of EPN and LPP effects, two streams of analysis were conducted. First, using fixed time windows, EPN and LPP amplitudes were scored as the mean activity in time windows of 192–252 and 380–480 ms, respectively. The second stream of analysis was based on individually adjusted EPN and LPP time windows (as described below), which is reported in the manuscript to align group and single‐subject analyses. However, the findings from both streams were highly similar.

Linear mixed models (LMM) were fitted based on EPN and LPP single trial data with a maximum likelihood (ML) estimator using R software (R Core Team, 2022) and the lme function from the “nlme” package (Pinheiro et al., 2023; see Supplemental Material S1 for the R‐code). To facilitate comparison of group and single subject analysis, separate models were computed for erotica and mutilation categories as well as for EPN and LPP components, respectively. Furthermore, a single subject analysis was conducted with the number of trials equated for conditions (see below) and LMMs were based on the same trials as analyzed in single subject analysis. However, repeating the analysis without this constraint confirmed the results reported in the manuscript. Missing trial data were not imputed.

All models included a random intercept and three fixed effects, Emotion (strong vs. weak), Time (trial 0 to 800) and the interaction of Emotion x Time, yielding the following formula (Wilkinson notation):

$$\text{Amplitude}\sim \text{Emotion}\;\text{*}\;\text{Time}+\left(1|\text{Participant}\right).$$

The factor Emotion was dummy‐coded (0 = strong; 1 = weak). All models used a first‐order autoregressive covariance structure (corAR1) with the order of observations serving as covariate and no assumed autocorrelation. Random slopes for Time (i.e., Time|Participant) were not included due to non‐convergence of the models. For fixed effects, regression coefficients were tested using t‐tests with 95% confidence intervals (CI). Amplitude values are reported as estimated marginal means with 95%‐CI using the “emmeans” package (Lenth, 2022).

Overall model fit is reported as log‐likelihood value (*LL*). The overall explained variance was estimated by conditional pseudo‐*R* ^2^ (Nakagawa et al., 2017), the variance explained by group factors (i.e., participants) is reported by the adjusted intraclass‐correlation (ICC). In addition, random effects were tested with a log‐likelihood ratio test (LRT) and effect size is reported in SD with 95%‐CI.

The four models (Erotica: EPN & LPP, Mutilation: EPN & LPP) converged with *LL* = −57203.02, −54205.30, −57396.28, and −58593.42, explaining a total variance of *R* ^*2*^ = .16, .05, .20, and .03, and a group level variance of ICC = .13, .04, .17, and .03. Furthermore, the intercept varied significantly across participants (Erotica EPN: SD = 1.48, CI_95_ = [1.09; 2.01], *χ* ^2^(2) = 2626.40, *p* < .0001; Erotica LPP: SD = 0.71, CI_95_ = [0.20; 2.51], *χ* ^2^(2) = 798.63, *p* < .0001; Mutilation EPN: SD = 1.80, CI_95_ = [1.30; 2.48], *χ* ^2^(2) = 3833.66, *p* < .0001; Mutilation LPP: SD = 0.70, CI_95_ = [0.49; 0.99], *χ* ^2^(2) = 513.57, *p* < .0001).

#### 2.5.2 Group analyses: Emotion and order effects

Previous research revealed an additional N‐1 effect associated with the preceding picture, which consisted of reduced EPN and LPP amplitudes to emotionally arousing (pleasant and unpleasant) N‐1 pictures (Flaisch, Junghöfer, et al., 2008; Flaisch, Stockburger, et al., 2008). Accordingly, a series of 3 × 2 repeated measures ANOVAs, including factors of N‐1 Category (erotica, neutral, and mutilation) and Emotion (erotica and neutral; mutilation and neutral), were conducted to explore possible order effects.

### 2.6 Single subject bootstrap analysis

EPN and LPP data from each individual case were submitted to a bootstrap analysis (Di Nocera & Ferlazzo, 2000; Efron & Tibshirani, 1993; Rosenfeld, 2020; Schupp & Kirmse, 2021). Specifically, with 50,000 bootstrap repetitions, each case's EPN and LPP mean data were resampled by randomly (re‐)assigning a case's trials to the categories of interest (i.e., erotica vs. neutral; mutilation vs. neutral) with replacement. The mean difference between the resulting ERPs was calculated for each bootstrap run.

To infer an effect common to the individuals comprising the study sample, the same sensor clusters for the EPN and LPP were used as in the group analyses. However, the latency of the components was allowed to vary between participants and emotion categories to acknowledge inter‐individual variability in functional brain organization. As in previous research (Schupp et al., 2023; Schupp & Kirmse, 2021, 2022), an effect for the EPN should appear between 150 and 350 ms and for the LPP between 350 and 750 ms. Within these temporal constraints, custom software identified the time window exhibiting the maximum EPN and LPP difference effect (high vs. low arousal) for each case. Specifically, a sliding window algorithm scored the mean activity using a 60 ms time window in the EPN cluster and a 100 ms time window in the LPP cluster. The time window showing the maximum negative (EPN) or positive (LPP) difference within their respective time boundaries was then selected for each individual case and used to calculate the single‐subject bootstrap distribution. Significance (*p* < .05, one‐sided) on the individual case level was determined as the proportion of results in the empirical probability distribution that were equal or more extreme than the de facto measured EPN/LPP difference. *p*‐values of *p* ≤ .05, *p* ≤ .01, *p* ≤ .001, and *p* < .00002, respectively, indicate that less than or equal to 2.500, 500, 50, and 0 out of 50.000 randomized calculations yielded an equal or more extreme result.

For exploratory reasons, we also assessed affective habituation effects. First, we computed the difference between the affective and neutral conditions at the single‐trial level. Preserving temporal order, EPN and LPP amplitudes were extracted for the erotic and mutilation conditions and then subtracted from the corresponding neutral conditions. Second, the resulting single‐trial difference vectors (high–low arousal) for the erotic and mutilation conditions were then separately analyzed using the Matlab function polyfit to determine linear trend effects over time. To calculate a bootstrap statistic, we randomly re‐shuffled the difference vector and calculated the linear trend.

## 3 RESULTS

### 3.1 Group analysis: Emotion and habituation

**FIGURE 1:**
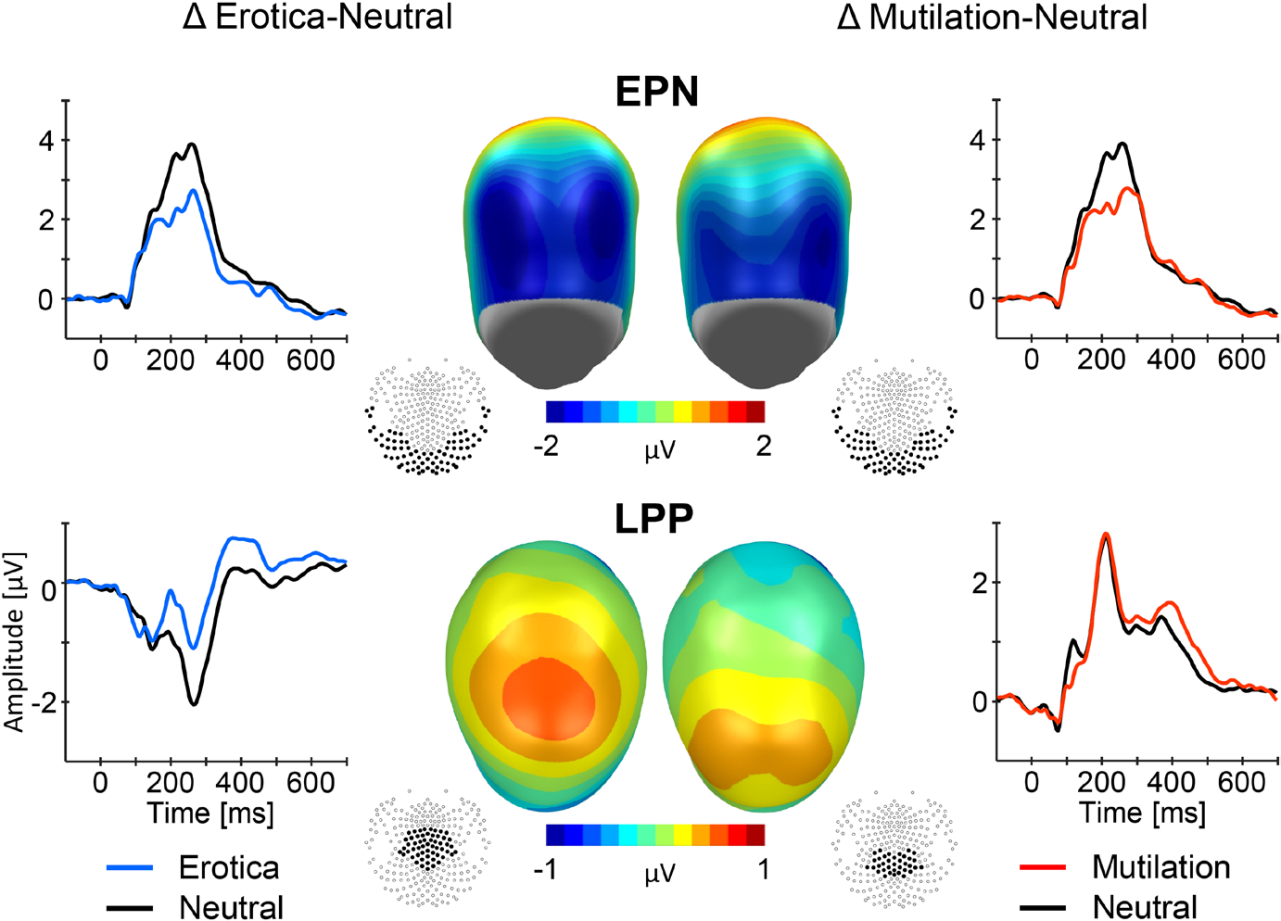
Illustration of event‐related brain potential (ERP) waveforms and difference scalp maps (high–low arousal) of the emotional modulation of the early posterior negativity (EPN) and late positive potential (LPP). Grand mean ERP waveforms were calculated using the respective clusters displayed in the sensor layouts (with the nose pointing up). The upper panels showing a back view of the model head illustrate the EPN findings which appear as a relative negative shift for high compared to low arousing images. The lower panels, showing a top view of the model head (with the nose pointing up), illustrate the LPP effect, appearing as a positive shift for highly arousing images. The scalp maps represent the average difference across a time window from 192–252 ms (EPN) and 380–480 ms (LPP).

**FIGURE 2:**
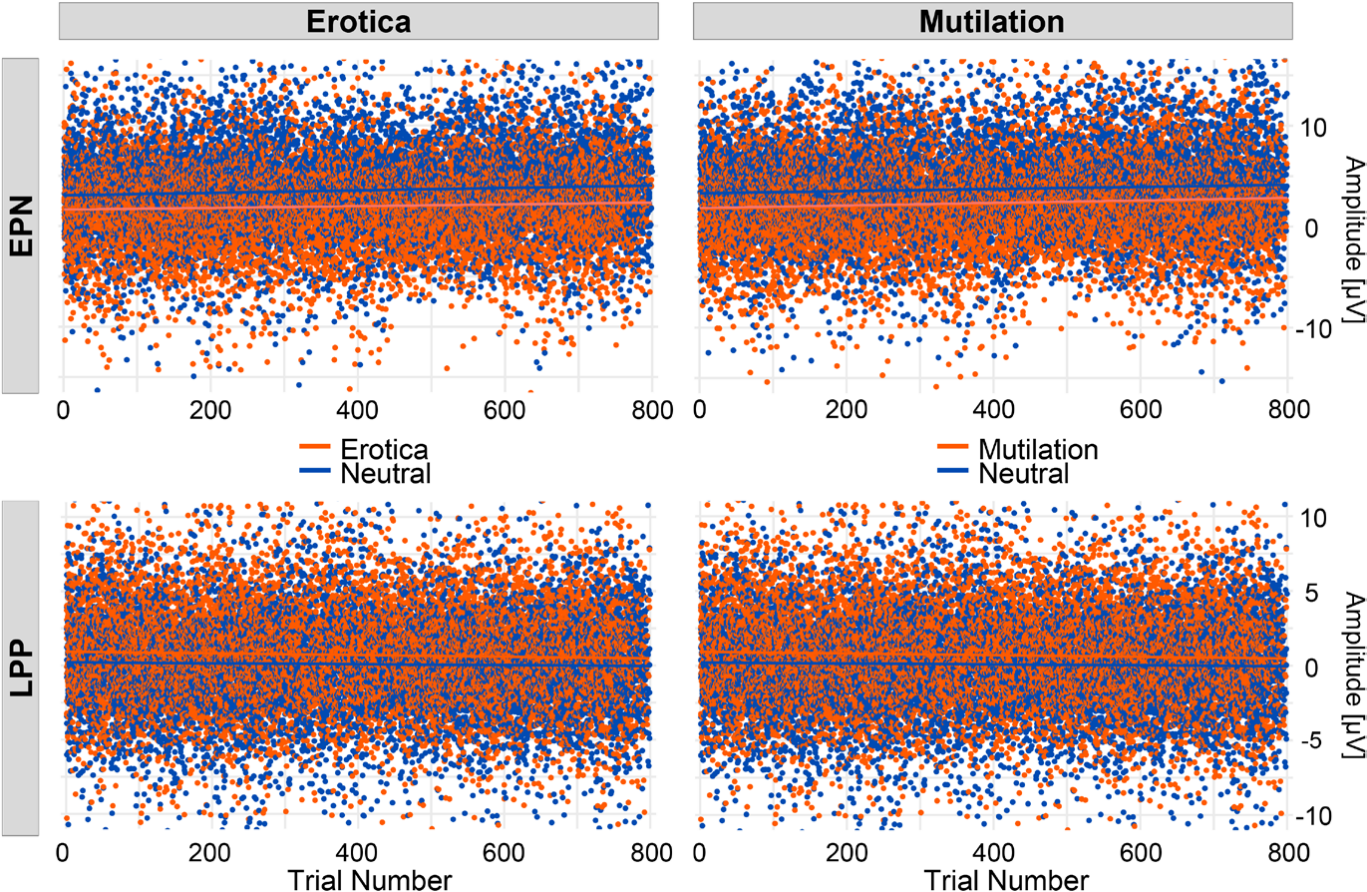
Emotional modulation of the early posterior negativity (EPN) and late positive potential (LPP) appeared stable across stimulus repetitions. Each colored dot represents the single trial EPN/LPP activity, averaged across participants. Colored lines represent a linear regression trend.

As shown in Figure 1, repeating the same erotic, neutral, and mutilation picture over and over again led to similar effects as observed previously using multiple exemplars in each stimulus category. Specifically, the processing of the erotic and mutilation picture was associated with enlarged EPN and LPP components as compared to the control stimulus. However, compared to previous research, the magnitude of the effect appeared somewhat reduced, particularly for the LPP component. Furthermore, while the topography of the EPN effect was similar for the erotic and mutilation picture, the LPP effect showed differences across conditions with a more centro‐parietal distribution for mutilations. Somewhat surprisingly, as shown in Figure 2, we observed no evidence of affective habituation effects. Specifically, neither the EPN nor the LPP components showed a decline in the emotional modulation effect across 800 repetitions of each image.

#### 3.1.1 Erotica

For the EPN, the erotic (*M* = 1.65 μV, CI_95_ = [0.86; 2.45]) as compared to the neutral picture (*M* = 3.23 μV, CI_95_ = [2.44; 4.03]) was associated with a relative negative potential difference (Δ*M* = −1.58 μV), Emotion (*b* = 1.39, *t*(20581) = 12.98, *p* < .0001, CI_95_ = [1.18; 1.60]). Furthermore, a significant effect of Time (*b* = 7.11 × 10^−4^, *t*(20581) = 4.21, *p* < .0001, CI_95_ = [3.80 × 10^−4^; 1.04 × 10^−3^]) showed a slightly increased overall amplitude over trials (*M*_0_ = 2.08 μV, *M*_800_ = 2.85, Δ*M* = 0.77 μV). The two‐way interaction of Emotion by Time was significant (*b* = 4.91 × 10^−4^, *t*(20581) = 2.07, *p* = .038, CI_95_ = [2.53 × 10^−5^; 9.56 × 10^−4^]), indicating an increase rather than a decrease of the emotional modulation over time.

For the LPP, a relative positive potential difference (Δ*M* = 0.61 μV) was observed for the erotic (*M* = 0.72 μV, CI_95_ = [0.34; 1.11]) as compared to the neutral image (*M* = 0.11 μV, CI_95_ = [−0.27; 0.50]), Emotion (*b* = −0.67, *t*(20581) = −7.29, *p* < .0001, CI_95_ = [−0.85; −0.49]). Furthermore, a significant effect of Time (*b* = −4.68 × 10^−4^, *t*(20581) = −3.20, *p* = .0014, CI_95_ = [−7.54 × 10^−4^; −1.81 × 10^−4^]) showed a slight amplitude decrease over trials (*M*_0_ = 0.57 μV, *M*_800_ = 0.26, Δ*M* = −0.31 μV). The two‐way interaction of Emotion by Time was not significant (*b* = 1.56 × 10^−4^, *t*(20581) = 0.77, *p* = .44).

#### 3.1.2 Mutilations

For the EPN, the mutilation (*M* = 2.22 μV, CI_95_ = [1.26; 3.18]) as compared to the neutral image (*M* = 3.68 μV, CI_95_ = [2.72; 4.64]) was associated with a relative negative potential shift (Δ*M* = −1.46 μV; Emotion: *b* = 1.61, *t*(20560) = 14.85, *p* < .0001, CI_95_ = [1.39; 1.82]). Furthermore, a significant effect of Time (*b* = 1.34 × 10^−3^, *t*(20560) = 7.87, *p* < .0001, CI_95_ = [1.00 × 10^−3^; 1.67 × 10^−3^]) showed a slight amplitude increase over trials (*M*_0_ = 2.51 μV, *M*_800_ = 3.42, Δ*M* = 0.91 μV). The two‐way interaction of Emotion by Time was not significant (*b* = −3.73 × 10^−4^, *t*(20560) = −1.56, *p* = .12).

For the LPP, processing of the mutilation (*M* = 1.11 μV, CI_95_ = [0.72; 1.49]) as compared to the neutral image (*M* = 0.54 μV, CI_95_ = [0.16; 0.92]) was associated with a relative positive potential difference (Δ*M* = 0.57 μV; Emotion: *b* = −0.68, *t*(20560) = −5.99, *p* < .0001, CI_95_ = [−0.91; −0.46]). Furthermore, a significant effect of Time (*b* = −4.04 × 10^−4^, *t*(20560) = −2.24, *p* = .025, CI_95_ = [−7.57 × 10^−4^; −5.08 × 10^−5^]) showed a slight amplitude decrease over trials (*M*_0_ = 0.92 μV, *M*_800_ = 0.72, Δ*M* = −0.20 μV). The two‐way interaction of Emotion by Time was not significant (*b* = 3.05 × 10^−4^, *t*(20560) = 1.20, *p* = .23).

### 3.2 Group analyses: Emotion and order effects

#### 3.2.1 Erotica

For the EPN, significant main effects of Emotion (*F*(1, 15) = 17.4, *p* < .001, $\eta_{p}^{2}$ = .538) and N‐1 Category (*F*(2, 30) = 20.9, *p* < .001, $\eta_{p}^{2}$ = .582) were observed. Replicating previous research (Flaisch, Junghöfer, et al., 2008; Flaisch, Stockburger, et al., 2008), post‐hoc testing indicated that emotional N‐1 pictures, that is, erotica (*M* = 3.01 μV, CI_95_ = [1.99; 4.03]) and mutilation (*M* = 3.16 μV, CI_95_ = [2.09; 4.23]), elicited a reduced EPN as compared to the neutral N‐1 image (*M* = 2.75 μV, CI_95_ = [1.68; 3.81]; *t*s ≥ 3.9, *p*s ≤ .001, Cohen's *d*s ≥ 0.98). The interaction Emotion by N‐1 Category was not significant (*F*(2, 30) = 0.32, *p* = .728).

For the LPP, the significant main effect of Emotion (*F*(1, 15) = 21.9, *p* < .001, $\eta_{p}^{2}$ = .593) was qualified by a significant interaction of Emotion by N‐1 Category (*F*(2, 30) = 5.15, *p* = .022, *ε* = 0.735, $\eta_{p}^{2}$ = .256). This effect appeared to be driven by reduced LPPs for repeated presentations of the erotic image. Post‐hoc testing revealed a significantly reduced LPP to the erotic‐erotic (*M* = 0.49 μV, CI_95_ = [0.11; 0.86]) as compared to the mutilation‐erotic (*M* = 0.71 μV, CI_95_ = [0.22; 1.19]) picture sequence (*t*(15) = −2.40, *p* = .030, uncorrected, Cohen's *d* = −0.60), and the comparison with the neutral‐erotic sequence approached significance (*M* = 0.71 μV, CI_95_ = [0.14; 1.28]); (*t*(15) = −1.64, *p* = .121). In contrast, there were no significant effects of the N‐1 category on neutral image processing (*t*s ≤ |1.46|. *p*s ≥ .166).

A further approach to explore the interaction of Emotion by N‐1 Category was to examine the emotional modulation of the LPP separately for the three N‐1 categories. Emotional modulation of the LPP was strong for mutilation and neutral N‐1 images (*t*s(15) ≥ 3.54, *p*s ≤ .003, Cohen's *d*s ≥ 0.88), and attenuated for erotic N‐1 images (*t*(15) = 2.19, *p* = .045, uncorrected, Cohen's *d* = 0.55).

#### 3.2.2 Mutilation

For the EPN, a similar pattern of finding emerged as for erotic stimuli. Specifically, significant main effects of Emotion (*F*(1, 15) = 33.4, *p* < .001, $\eta_{p}^{2}$ = .690) and N‐1 Category (*F*(2, 30) = 7.2, *p* = .003, $\eta_{p}^{2}$ = .323) were observed, while the interaction Emotion by N‐1 Category was not significant (*F*(2, 30) = 1.1, *p* = .357). Follow‐up tests of the N‐1 Category effect revealed that the mutilation N‐1 picture (*M* = 3.16 μV, CI_95_ = [2.12; 4.20]) led to a reduced EPN as compared to the neutral N‐1 image (*M* = 2.86 μV, CI_95_ = [1.85; 3.87]; *t*(15) = 4.00, *p* = .001, Cohen's *d* = 1.00) while the according comparison with the erotic N‐1 image (*M* = 3.01 μV, CI_95_ = [1.98; 4.02]) was not significant (*t*(15) = 1.65, *p* = .119).

For the LPP, the significant main effect of Emotion (*F*(1, 15) = 13.4, *p* = .002, $\eta_{p}^{2}$ = .472) was qualified by a significant interaction of Emotion by N‐1 Category (*F*(2, 30) = 9.50, *p* < .001, $\eta_{p}^{2}$ = .388). Following up on this interaction, the positivity to the mutilation‐mutilation picture pair (*M* = 1.06 μV, CI_95_ = [0.64; 1.48]) was reduced compared to the neutral‐mutilation picture sequence (*M* = 1.54 μV, CI_95_ = [1.04; 2.05]; *t*(15) = −3.41, *p* = .004, Cohen's *d* = −0.85). The comparison with the erotic‐mutilation pair (*M* = 1.36 μV, CI_95_ = [0.88; 1.83]) approached significance (*t*(15) = −1.89, *p* = .079, Cohen's *d* = −0.47). Furthermore, neutral picture processing was also associated with reduced positivity when following a neutral (*M* = 0.67 μV, CI_95_ = [0.25; 1.08]) as compared to either the erotic (*M* = 0.99 μV, CI_95_ = [0.53; 1.45]; *t*(15) = −3.01, *p* = .009, Cohen's *d* = −0.75) or mutilation N‐1 picture (*M* = 1.01 μV, CI_95_ = [0.55; 1.48]; *t*(15) = −3.09, *p* = .007, Cohen's *d* = −0.77).

Testing the emotional modulation of the LPP separately for the three N‐1 images revealed a strong emotional modulation for the neutral N‐1 image (*t*(15) = 5.17, *p* < .001, Cohen's *d* = 1.29), and the emotional modulation effect approached significance for the erotic N‐1 image (*t*(15) = 2.07, *p* = .056, Cohen's *d* = 0.52). Conversely, no effect was observed for the mutilation N‐1 image (*t*(15) = 0.37, *p* = .720), indicating a strong repetition effect for the mutilation image.

#### 3.2.3 Affective habituation

We also conducted exploratory analyses to assess the effects of the N‐1 picture on affective habituation by calculating separate LLMs for each of the three N‐1 pictures. The findings from these separate models aligned with those of the overall models, indicating internal consistency (see Supplemental Material S2).

### 3.3 Single subject analysis

**FIGURE 3:**
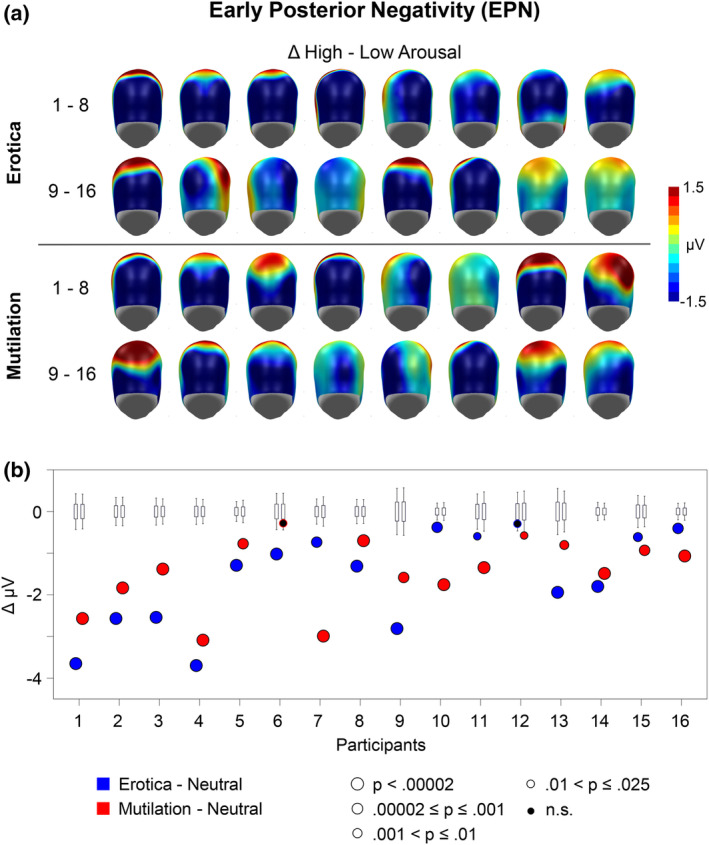
Illustration of the single subject findings for the early posterior negativity (EPN). (a) The upper panel shows difference scalp maps (high‐low arousal) of the emotional modulation of the EPN for each individual case. A back view of the model head is displayed. Maps show the individual time window of the maximal EPN difference as selected by the automatic software routine. Please note the common scale (±1.5 μV), which leads to truncation of effects in individuals showing large effects. (b) The lower panel illustrates the case‐by‐case statistics. The bootstrap distribution is shown for each case in a boxplot. The bottom and top edges of the box indicate the 25th and 75th percentiles while the whiskers indicate the 5th and 95th percentiles. The dots illustrate the de facto measured EPN difference (high–low arousal). Dots outside the range indicated by the whiskers represent significant effects (*p* < .05) with size and color indicating different *p*‐levels.

**FIGURE 4:**
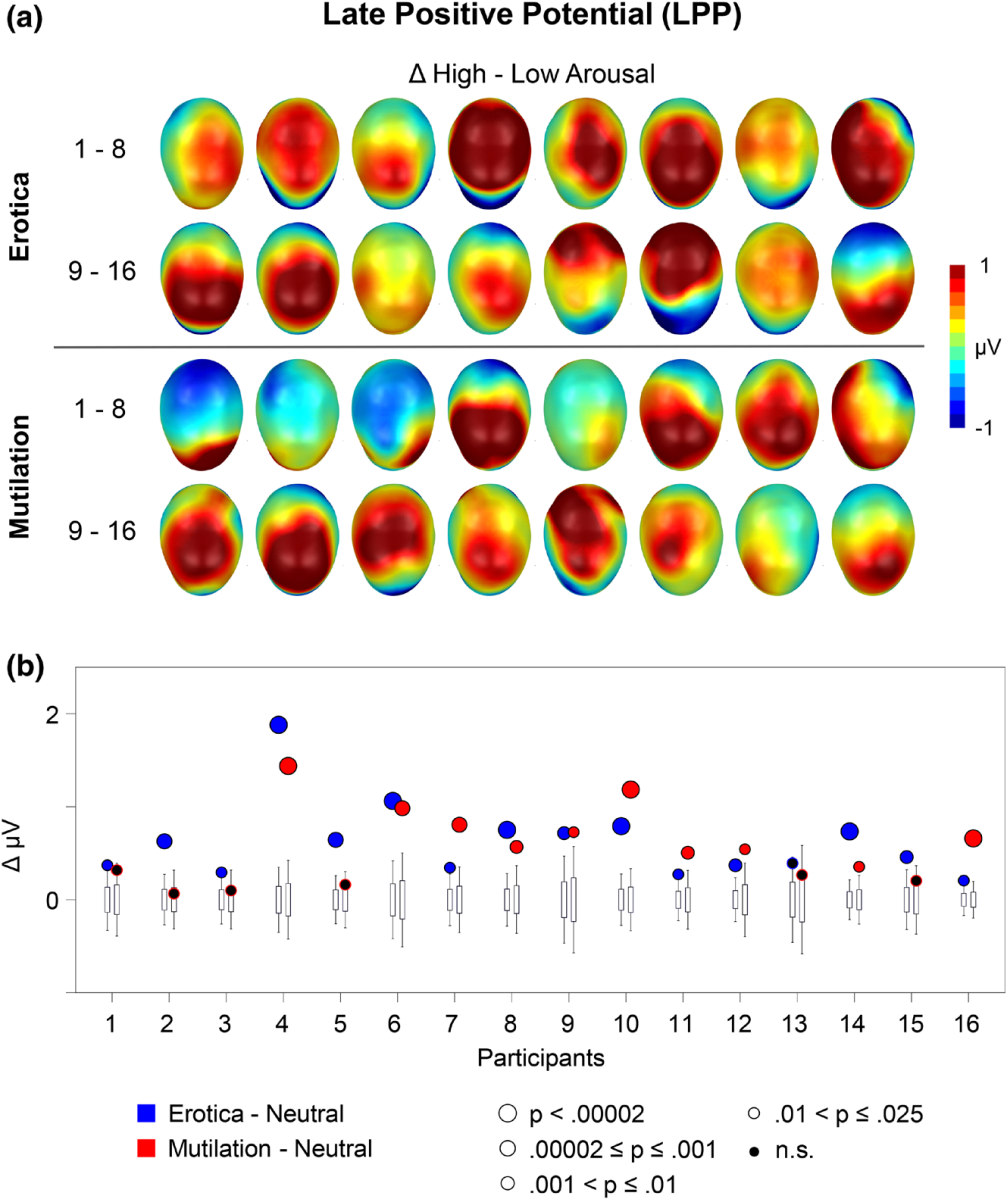
Illustration of the single subject findings for the late positive potential (LPP). (a) The upper panel shows difference scalp maps (high‐low arousal) of the emotional modulation of the LPP for each individual case. A top view of the model head is displayed (with the noise pointing up). (b) The lower panel illustrates the case‐by‐case statistics. For more details, please refer to Figure 3.

Findings from single subject analysis are shown in Figure 3 (EPN) and Figure 4 (LPP). The upper panel of each figure shows the emotional modulation effect observed for each individual case by displaying the difference scalp maps for the high‐arousing erotic and mutilation pictures as compared to the low arousing neutral image. A common scale was used to display the effects, which led to truncation of the data in individual cases with large effects. The lower panel illustrate the bootstrap findings by displaying the measured EPN difference, the observed p‐level of significance, and the random‐resampling bootstrap distribution.

#### 3.3.1 Erotica

For the EPN, 15 out of 16 participants (94%) showed significantly larger EPN amplitudes for the erotic compared to the neutral control picture. Similarly, 15 participants (94%) showed a significantly larger LPP component for the erotic than neutral image.

#### 3.3.2 Mutilation

For the EPN, findings for the mutilation and erotic picture were similar. Specifically, 15 out of 16 participants (94%) showed significantly larger EPN amplitudes for the mutilation compared to the neutral picture. However, for the LPP, the single subject statistics appeared more variable including displays of small to no effect in several cases. Specifically, significantly larger LPPs to the mutilation than to the neutral image was observed in only 10 out of 16 cases (63%).

#### 3.3.3 Affective habituation effects

While group analysis showed no evidence for affective habituation effects, exploratory analyses were undertaken at the single subject level. For the EPN, two and one cases showed a significant decrease of the emotion effect across time for either the erotica or mutilation images, respectively. However, another three cases showed a slight increase in the emotion effect across time in the mutilation condition. For the LPP, one case showed affective habituation effects for either the erotic or mutilation condition, respectively.

#### 3.3.4 Emotion and task effects as a function of trial number

**FIGURE 5:**
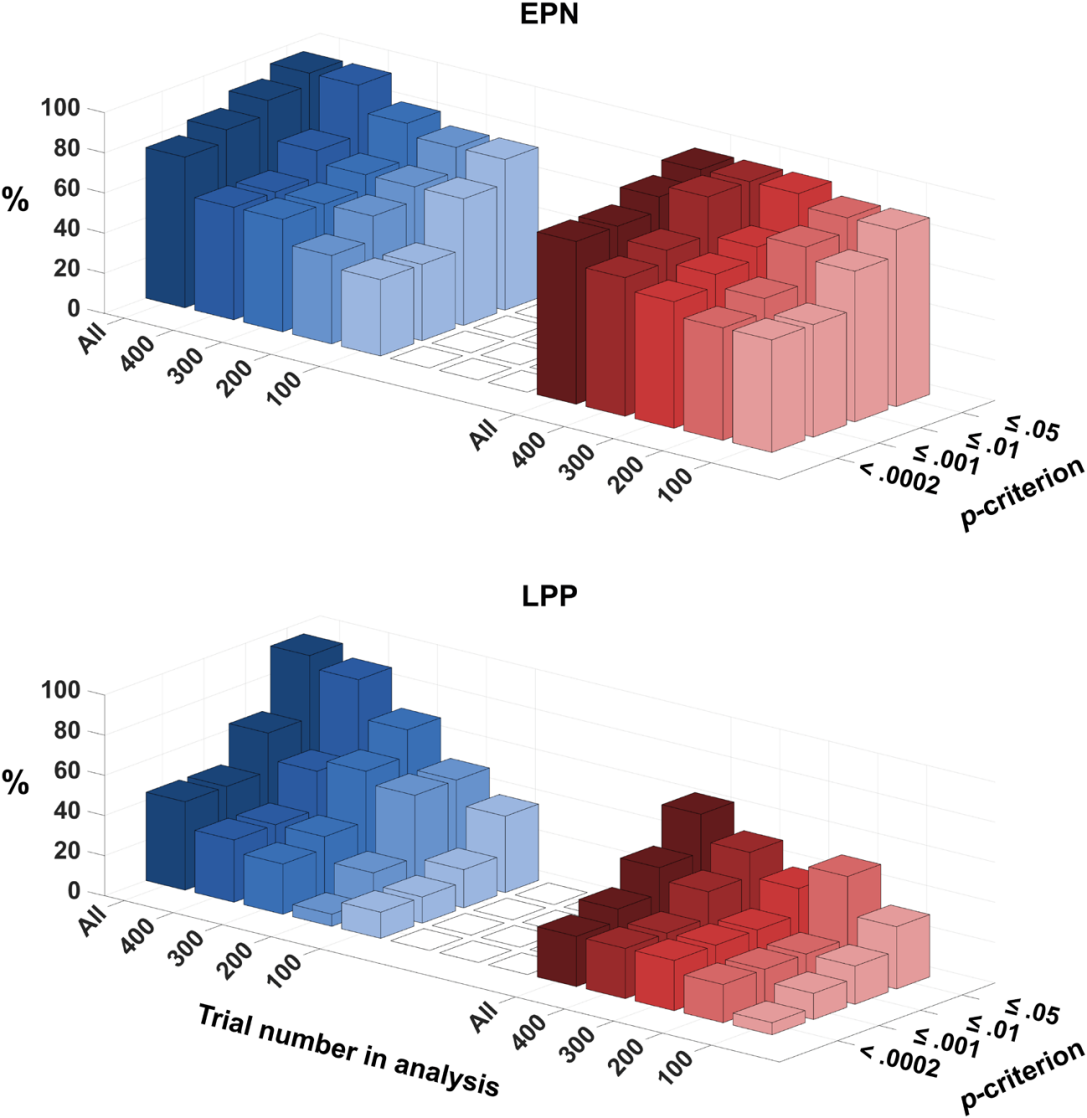
Proportion (%) of significant cases as a function of *p*‐ criterion and number of trials used for the calculation of the bootstrap sampling distribution. Blue and red colors indicate the comparison of erotica‐neutral and mutilation‐neutral, respectively.

Analyses progressively down‐sampling the number of trials used to calculate the bootstrap statistic were conducted to assess the proportion of significant single‐subject tests as a function of trial number, that is, using the first 400, 300, 200, and 100 trials per condition (total *N* = 800, 600, 400, 200, and 100, respectively). As shown in Figure 5, trial number had differential effects on the emotional modulation of the EPN and LPP. Considering the *p* ≤ .05 criterion, there was some decline in the proportion of significant tests with decreasing trial number for the erotic image, that is, dropping from 94 to 75%, while the effect appeared to be stable for the mutilation image, that is, ≥88%. A rather different picture emerged for the LPP. For both picture categories, the proportion of significant tests dropped considerably with decreasing trial numbers, reaching significance in 38% and 31% of cases for the erotic and mutilation images, respectively. Furthermore, the proportion of significant tests progressively declined as the number of trials decreased, particularly when stricter p‐criteria were applied.

#### 3.3.5 Control analyses: Picture order

To take effects associated with the order of picture presentation into account, control analyses were calculated determining the emotional modulation effects separately for the three different N‐1 pictures.

For the EPN, effects appeared similar across the three N‐1 picture contents. Specifically, significantly enlarged EPN amplitudes to the erotic compared with the neutral image was observed in 13, 13, and 14 out of 16 participants when analyzed separately for erotic, neutral, and mutilation images shown in the preceding trial. Similarly, for erotic, neutral, and mutilation N‐1 images, the emotional EPN modulation for the mutilation image was observed in 13, 13, and 15 cases.

A more nuanced pattern of findings emerged for the LPP component, with differential effects depending on the content of the N‐1 picture. When the N‐1 trials showed neutral images, emotional modulation of the LPP was observed in 12 out of 16 cases for both the erotic and mutilation conditions. However, when the N‐1 trial presented a different emotional image—such as in N‐1—N sequences of mutilation‐erotic versus mutilation‐neutral, and erotic‐mutilation versus erotic‐neutral—the effect reached significance in 13 and 8 cases for erotica and mutilation, respectively. Conversely, when the N‐1 trial displayed the same emotional image—such as in sequences of erotic‐erotic versus erotic‐neutral, and mutilation‐mutilation versus mutilation‐neutral—the effect reached significance in only five and four cases for erotica and mutilation, respectively.

## 4 DISCUSSION

The present study aimed to assess effects of emotional modulation and affective habituation of the EPN and LPP components within a one‐picture‐per‐emotion‐category protocol. Regarding emotional modulation, group analyses revealed significantly larger EPN and LPP components for the high‐arousing erotic and mutilation image compared to the low‐arousing neutral control stimulus. These findings motivated a case‐by‐case analysis to determine how many participants of the study sample show the effect at the individual level. For the EPN, 15 out of 16 participants showed the effect for the erotic as well as the mutilation category. For the LPP, a more nuanced pattern of findings emerged: While the erotic category reached significance in 15 out of 16 cases, the effect was significant in only 10 cases for the mutilation category. With regard to affective habituation, there was no significant decline of the emotional differentiation of the EPN and LPP components across time at the group level and this pattern of finding was confirmed by analyzing individual cases. Overall, these findings show that emotion effects can reliably be observed for a singular stimulus and encourage the pursuit of an individualized approach to study the process of affective stimulus evaluation.

### 4.1 Emotional modulation effects: Group analyses

A first stream of analysis confirmed EPN and LPP effects to singular affective stimuli at the group level. Robust emotional modulation effects were observed for the EPN component with erotic and mutilation images prompting a sizable negative difference potential (Δ: −1.58 and −1.46 μV) over posterior sensor sites as compared to a neutral image. A significant emotional modulation also emerged for the LPP potential. However, the size of the emotional modulation of the LPP appeared attenuated (Δ: 0.61 and 0.57 μV for erotic and mutilation images). This may be explained by massive stimulus repetition, that is, 800 presentations of each image, strongly reducing novelty of the images compared to research paradigms using multiple exemplars per stimulus category (see Ferrari et al., 2022). However, the lack of affective habituation effects, along with the observation that reducing the trial number to 100 trials per category still resulted in a substantial proportion of participants showing a significant emotional modulation of the EPN to erotica (75%) and mutilation (88%) pictures constrains this interpretation. Overall, as reflected by the EPN and LPP components, emotional modulation to singular erotic and mutilation images was reliably observed at the group level.

The differences in findings between one‐ and many‐picture‐per‐emotion‐category designs merit further consideration. While there is evidence that emotional modulation effects can be obtained even when controlling for low‐level physical stimulus characteristics (Junghöfer et al., 2001), challenges in selecting stimulus materials increase when using only one compared to many exemplars per emotion category. For instance, the topography of the emotional modulation of the LPP was rather different for the mutilation compared to the erotic image, and, in comparison to the topography observed in previous research regarding mutilations (Hajcak et al., 2010; Lang & Bradley, 2010; Schupp, Flaisch, et al., 2006). This may reflect the particular stimuli chosen to represent the emotion categories or may result from using a singular control stimulus rather than matching each emotional stimulus to a corresponding neutral exemplar, which may provide better control over figure‐ground composition and other physical stimulus characteristics (Bradley et al., 2007; De Cesarei & Codispoti, 2013; Junghöfer et al., 2000). It might be informative to systematically compare the processing of the same stimulus materials when presented either in a one‐picture‐per‐emotion‐category protocol or embedded within a stream of pictures showing multiple different exemplars. Furthermore, systematic variations of a picture with regard to the prototypicality of stimulus features may provide unique insights into which stimulus characteristics drive emotional EPN and LPP effects (Farkas & Sabatinelli, 2023).

The present findings also indicate effects attributable to the N‐1 picture category. For the EPN, a main effect of the N‐1 trial category has been observed in previous research, where pleasant and unpleasant N‐1 picture categories, compared to neutral ones, were associated with a reduced EPN component (Flaisch, Junghöfer, et al., 2008; Flaisch, Stockburger, et al., 2008). This effect was replicated in the present study, with three out of four independent tests reaching significance and the remaining one approaching significance. A more complex pattern of findings emerged for the LPP. Previous findings of a reduced LPP component for emotionally arousing images (pleasant and unpleasant) were not replicated in the present study (Flaisch, Stockburger, et al., 2008). Instead, the findings suggest an N‐1 effect associated specifically with the repetition of the same picture, irrespective of picture content. However, this effect did not consistently reach significance in the present study, resonating with previous research on stimulus repetition effects (Ferrari et al., 2015). The hypothesis that the repetition of stimuli is associated with a reduced positivity, potentially reflecting suppression effects (Ferrari et al., 2015), awaits further research.

### 4.2 Emotional modulation effects: Case‐by‐case analyses

Significant group findings provided the foundation for the analysis of the individual case. A robust and consistent finding emerged for the EPN component: 15 out of 16 participants showed a significant EPN effect for the erotic as well as the mutilation stimulus category. Interestingly, the two non‐significant effects were shown by different individuals, suggesting a lack of emotional modulation to a specific stimulus category rather than a general insensitivity to affective stimulus significance indicated by the EPN component (see also Schupp & Kirmse, 2022). Overall, with regard to the EPN component, the percentage of cases showing significant effects in the present one‐picture‐per‐emotion‐category study is similar to previous studies presenting multiple exemplars per stimulus category (Schupp et al., 2023; Schupp & Kirmse, 2021, 2022).

The case‐by‐case analysis of the LPP component yielded a more nuanced pattern of results. All but one individual showed a significantly enlarged LPP to the erotic image (94%) resonating with previous findings using multiple stimuli per category (Schupp et al., 2023; Schupp & Kirmse, 2021, 2022). However, findings for the mutilation category were less consistent. Only 10 out of 16 cases showed a significantly larger LPP to singular mutilation images, significance was approached in another case, while 5 cases showed no effect. Thus, the percentage of cases showing a significant LPP effect is substantially lower compared to previous research (63% vs. 98%). The differences in the number of cases showing significant LPP modulations for erotica and mutilations need to be resolved in future research. For instance, there remains the possibility that individuals showing non‐significant LPP effects avoided to attentively look at displays of mutilation. Implementing an active task may address this concern. The feasibility of this approach has been demonstrated in a recent study in which participants categorized emotional and neutral stimuli. Behavioral performance in this task, as well as assessment of the P3 target effect allowed to determine whether participants actually did process the pictures (Schupp et al., 2023).

The finding that emotional modulation effects may vary depending on the previously seen picture motivated a series of control‐analyses assessing the emotional modulation of the EPN and LPP separately for the three different N‐1 pictures. For the EPN, the N‐1 picture had little impact on the proportion of cases showing significantly larger EPN amplitudes to erotic and mutilation images as compared to the neutral control picture, that is, ≥81% in six independent analyses. A different pattern of findings emerged for the LPP. Here, the three different N‐1 pictures had a profound effect on the number of cases showing emotional modulation effects. For the neutral N‐1 picture, 75% of the tests reached significance for both the erotic and mutilation images. For the emotional N‐1 picture showing opposite valence to the test picture, that is, an N‐1 mutilation image when exploring an erotic LPP effect and vice versa, 81% and 50% of the cases reached significance for erotic and mutilation images, respectively. In contrast, when the N‐1 image had the same valence as the test picture, only 31% and 25% of the tests reached significance. These findings demonstrate that stimulus repetition has a suppressive effect on case‐wise emotional modulation of the LPP. Therefore, experimental protocols designed to probe the idiosyncratic nature of emotions may require the presentation of an irrelevant picture in future research to mitigate stimulus repetition effects.

The present findings have implications for studying the idiosyncratic nature of emotion and the translation of basic research to the clinical domain. The findings suggest that it is possible to assess early processes of affective stimulus evaluation within a one‐picture‐per‐emotion‐category design at the level of the individual case. This provides the foundation for selecting personally meaningful stimulus materials and anchoring them within a space defined by the processing of emotionally significant stimuli shared across individuals. More specifically, presenting prototypical stimuli from evolutionarily relevant behavior domains alongside individually tailored images may be a promising way to map individual emotional landscapes. From a translational perspective, changes in the emotional modulation of the EPN and LPP were observed for various mental disorders, for example, psychopathy, depression, generalized anxiety disorder, and trauma‐related psychopathology (Klawohn et al., 2021; MacNamara et al., 2016; Sill et al., 2020; Vallet et al., 2020). It seems possible to extend the group perspective to the individual case using idiosyncratic stimulus materials. In short, affective stimulus evaluation as a process common‐to‐all can shed light on the complex idiosyncratic nature of emotion (Frijda, 1988).

### 4.3 Affective habituation: Group and case‐by‐case analyses

The present study extended the analysis of affective habituation through the massive repetition of one picture per category. With multiple exemplars representing stimulus categories, previous research showed either no or small affective habituation effects of the EPN and LPP component (Codispoti et al., 2016; Ferrari et al., 2020, 2022; Mastria et al., 2017; Schupp, Stockburger, et al., 2006). The present findings align with these observations. Specifically, at the group level, linear mixed modeling analyses indicated no decline in the emotional modulation of the EPN and LPP components, neither for the erotic nor mutilation image. Findings from the overall analyses were also confirmed when analyzing the data separately for the three N‐1 pictures. At the single case level, habituation was assessed by fitting linear trends on EPN and LPP amplitude differences (erotic/mutilation—neutral) over time, that is, over 800 repetitions, and in each person. For the EPN, only 2 (Δ erotica—neutral) and 1 (Δ mutilation—neutral) out of 16 tests provided evidence for affective habituation. In the mutilation condition, three cases even showed an increase of emotional modulation across time. Similarly, for the LPP, only one case for erotic and mutilation tests showed affective habituation effects. Overall, despite the massive repetition of a single picture representing each emotion category, the emotional modulation of the EPN and LPP did not vary over time.

### 4.4 Summary

The present study explored emotional modulation and habituation effects through the repeated presentation of a single erotic, mutilation, and neutral picture. Emotional modulation effects of the EPN and LPP components were observed at both the group and individual levels, with no decline in effects over time. Demonstrating that emotion effects can reliably be observed for singular stimuli may encourage the pursuit of an individualized approach to study the process of affective stimulus evaluation.

## AUTHOR CONTRIBUTIONS

**Harald T. Schupp:** Conceptualization; formal analysis; funding acquisition; investigation; methodology; software; supervision; visualization; writing – original draft; writing – review and editing. **Karl‐Philipp Flösch:** Formal analysis; methodology; software; visualization; writing – review and editing. **Tobias Flaisch:** Formal analysis; investigation; methodology; software; visualization; writing – review and editing.

## FUNDING INFORMATION

This research was supported in part by the Deutsche Forschungsgemeinschaft (DFG, German Research Foundation) under Germany's Excellence Strategy—EXC2117‐422037984 granted to Harald Schupp.

## CONFLICT OF INTEREST STATEMENT

The authors declare no conflicts of interest.

## Supporting information


**Data S1.** Supplemental Material S1. Commented R‐code for linear mixed models.
Supplemental Material S2. Linear mixed models for N‐1 analysis.

## Data Availability

The data have not been made available on a permanent third‐party archive because we do not have informed consent from participants to share their raw EEG data. However, the aggregated data supporting the findings of this study are available from the corresponding author upon reasonable request.

## Footnotes

^1^
Pleasant: 1610, 1710, 2080, 4290, 4460, 4606, 4610, 4652. Neutral: 1620, 1812, 2210, 2440, 2510, 2530, 2850, 4605. Unpleasant: 1301, 2900, 3015, 3063, 3120, 6250, 6550, 6313, 9570.

^2^
To be able to assess affective dishabituation, the complementary picture set was presented in trials 2401–2700. However, no affective habituation effects were observed in the analyses of trials 1–2400. The analyses of the dishabituation trials confirmed emotional modulation effects of the EPN (*t*s(15) ≤ −3.5, *p*s ≤ .003, *d*s ≤ −0.88) and LPP for erotica and mutilation compared to neutral pictures, (*t*s(15) ≥ 2.3, *p*s ≤ .031, *d*s ≥ 0.59).
