# Supplementary material for: A case‐by‐case analysis of EPN and LPP components within a “one‐picture‐per‐emotion‐category” protocol
Source: Psychophysiology. 2024 Nov 12;62(2):e14718. doi: 10.1111/psyp.14718 (PMC11870814; doi:10.1111/psyp.14718)
Supplement: Supplementary file 1 — Data S1. Supplemental Material S1. Commented R‐code for linear mixed models. Supplemental Material S2. Linear mixed models for N‐1 analysis. [file PSYP-62-e14718-s001.pdf]

Supplemental Material S1 – Commented R-code for linear mixed models

The following code was used to calculate linear mixed models (LMMs) on trial-wise EEG data for each participant and condition using the following packages.

Requirements

```
library(readr) # for importing data
library(dplyr) # for data handling
library(nlme) # for calculating LMMs
library(MuMIn) # for calculting Nakagawa's pseudo-R²
library(ggeffects) # for extracting cell means
library(performance) # for checking quality of model fit
library(reghelper) # for calculating model ICC
library(sjPlot) # for printing
```

Data format

The data were in long format, with the number of rows equal to the number of subjects × the number of trials per condition × the number of conditions (16 × 800 × 2). For each trial ('time'), there is a measurement variable ('amplitude') and two factor variables: the subject identifier ('subject') and the experimental condition ('emotion').

```
dataMat <- read_delim("D:/YourPath/TrialData.txt", "\t", escape_double = FALSE,
trim_ws = TRUE) # read from tsv file
```

LLM calculation

To calculate the models, the condition factor was linearly recoded.

```
dataMat$emotion.d <- recode(dataMat$emotion, "low" = 0, "high" = 1) # dummy coding
```

The LMM is calculated using a maximum likelihood (ML) estimator with the 'lme' function from the 'nlme' package. Although restricted ML (REML) estimators sometimes provide a better model fit — such as those used in the 'lme4' package — ML estimators allow for cross-model and cross-study comparisons of LMMs. A first-order autoregressive covariance structure (corAR1) was used to estimate a growth model with an equally spaced time vector (which approximately holds true for our trials). Missing data were not substituted (na.action = na.omit), since, unlike simple linear regression, LMMs can handle missing values. In a first step, we included random slopes and intercepts for each participant and condition. However, due to low model fit, models did not converge. Therefore, we restricted the analysis to random intercept models.

```
# model with random intercepts for participants
RegModel <- lme(amplitude ~ emotion.d*time, data = dataMat, random = ~ 1|subject,
method = 'ML', na.action = na.omit, correlation = corAR1(0, form = ~1|subject))

# model with random random slopes for participants (random intercepts are implied
by the term emotion.d*time|subject)
RegModel <- lme(amplitude ~ emotion.d*time, data = dataMat, random = ~
emotion.d*time|subject, method = 'ML', na.action = na.omit, correlation =
corAR1(0, form = ~1|subject))

# check for model fit
performance::check_singularity(RegModel) # getting FALSE is a good sign, we want
NO singularity

# print the model output
summary(RegModel)
coef(RegModel) # intercepts and slopes for each case
intervals(RegModel) # confidence intervals for fixed and random effects
ggemmeans(RegModel,'emotion.d') # extracting cell means for a given effect is most
easy using the 'ggeffects' package
```

Effect sizes

For the model effect size, we calculated Nakagawa's pseudo-R^2 to estimate the overall explained variance (random + fixed effects). The adjusted intraclass correlation coefficient (*ICC*) was calculated to estimate the variance explained by the group (random) effects.

```
# get model R² and ICC
r.squaredGLMM(RegModel) # Nakagawa's conditional pseudo-R² (= random + fixed
effects)
reghelper::ICC(RegModel) # adjusted ICC (= only random effects, see also
performance::icc)
sjPlot::tab_model(RegModel) # nice html print of model
```

In addition, we tested each random effect using a log-likelihood ratio test (*LRT*) via the 'anova' function, comparing the base model (with only fixed effects) to the updated model that includes the random effect in question. Random effect sizes are reported in standard deviations (*SD*), along with 95% confidence intervals (*CI*).

```
# Example: test random intercept
basicModel <- gls(amplitude ~ emotion.d*time, data = dataMat, method = 'ML',
na.action = na.omit) # linear model with fixed intercept
randModel <- lme(amplitude ~ emotion.d*time, data = dataMat, random = ~ 1|subject,
method = 'ML', na.action = na.omit, correlation = corAR1(0, form = ~1|subject))  #
LMM with random intercept
anova(basicModel,randModel) # comparing the -2LL of both models using LRT
intervals(RegModel) # get the SD with CI for random intercept of main model
```

We also explored effect sizes for fixed effects by calculating Cohen's f^2 as the ratio of explained variance from a reduced model (not including the fixed effect in question) to that of the full model (see Selya et al., 2012). Cohen's f^2 was calculated using the formula $f^2 = \frac{R^2_{full} - R^2_{red}}{1 - R^2_{full}}$.

```
# Example: Effect size factor 'emotion'
redModel <- lme(amplitude ~ time, data = dataMat, random = ~ 1|subject, method =
'ML', na.action = na.omit, correlation = corAR1(0, form = ~1|subject)) # LMM
without emotion
fullModel <- lme(amplitude ~ emotion.d*time, data = dataMat, random = ~ 1|subject,
method = 'ML', na.action = na.omit, correlation = corAR1(0, form = ~1|subject))  #
LMM with emotion
R2_withoutEmo tion <- r.squaredGLMM(redModel)[2] # conditional pseudo-R² without
emotion
R2_Full <- r.squaredGLMM(fullModel)[2] # conditional pseudo-R² with emotion
f2_Emotion <- (R2_Full-R2_withoutEmotion)/(1-R2_Full) # calculate Cohen's f²
```

References

Selya, A. S., Rose, J. S., Dierker, L. C., Hedeker, D., & Mermelstein, R. J. (2012). A practical guide to calculating Cohen'sf 2, a measure of local effect size, from PROC MIXED. *Frontiers in psychology*, *3*, 111. https://doi.org/10.3389/fpsyg.2012.00111

Supplemental Material S2 – Linear mixed models for N-1 analysis

We conducted exploratory analyses examining the effects of the three N-1 picture types on affective habituation. Specifically, we calculated separate linear mixed models (LLMs) for each ERP component, N category (erotic vs. neutral; mutilation vs. neutral), and N-1 picture category, respectively.

Early posterior negativity: Erotic vs. Neutral

N-1: Erotic

	Amplitude		
Predictors	***Estimates***	***CI***	***p***
(Intercept)	1.6779	0.9864 – 2.3694	**<0.001**
Emotion	1.2348	0.8732 – 1.5964	**<0.001**
Time	0.0007	-0.0012 – 0.0025	0.470
Emotion x Time	0.0028	0.0003 – 0.0054	**0.031**

Random Effects: Marginal R^2 = .039; Conditional R^2 = .140

N-1: Neutral

	Amplitude		
Predictors	***Estimates***	***CI***	***p***
(Intercept)	1.0483	0.2880 – 1.8087	**0.007**
Emotion	1.6992	1.3269 – 2.0716	**<0.001**
Time	0.0028	0.0009 – 0.0047	**0.004**
Emotion x Time	-0.0004	-0.0031 – 0.0022	0.745

Random Effects: Marginal R^2 = .044; Conditional R^2 = *NA**

N-1: Mutilation

	Amplitude		
Predictors	***Estimates***	***CI***	***p***
(Intercept)	1.4641	0.5451 – 2.3830	**0.002**
Emotion	1.5055	1.1383 – 1.8727	**<0.001**
Time	0.0038	0.0019 – 0.0057	**<0.001**
Emotion x Time	0.0013	-0.0013 – 0.0039	0.331

Random Effects: Marginal R^2 = .050; Conditional R^2 = *NA**

Early posterior negativity: Mutilation vs. Neutral

N-1: Erotic

	Amplitude		
Predictors	***Estimates***	***CI***	***p***
(Intercept)	1.5808	0.6647 – 2.4968	**0.001**
Emotion	1.6607	1.2968 – 2.0247	**<0.001**
Time	0.0043	0.0025 – 0.0061	**<0.001**
Emotion x Time	-0.0009	-0.0034 – 0.0017	0.507

Random Effects: Marginal R^2 = 0.044; Conditional R^2 = *NA**

N-1: Neutral

	Amplitude		
Predictors	***Estimates***	***CI***	***p***
(Intercept)	1.6511	0.7495 – 2.5527	**<0.001**
Emotion	1.5199	1.1489 – 1.8909	**<0.001**
Time	0.0042	0.0024 – 0.0061	**<0.001**
Emotion x Time	-0.0016	-0.0042 – 0.0010	0.229

Random Effects: Marginal R^2 = 0.032; Conditional R^2 = *NA**

N-1: Mutilation

	Amplitude		
Predictors	***Estimates***	***CI***	***p***
(Intercept)	1.8793	0.9436 – 2.8150	**<0.001**
Emotion	1.4320	1.0682 – 1.7958	**<0.001**
Time	0.0033	0.0015 – 0.0051	**<0.001**
Emotion x Time	0.0008	-0.0017 – 0.0034	0.528

Random Effects: Marginal R^2 = 0.043; Conditional R^2 = *NA**

* If no conditional R^2 is reported, this indicates that the model did not converge, likely due to an insufficient number of observations, and consequently includes no random but only fixed effects.

Late Positive Potential: Erotic vs. Neutral

N-1: Erotic

		Amplitude	
Predictors	***Estimates***	***CI***	***p***
(Intercept)	0.6252	0.2446 – 1.0058	**0.001**
Emotion	-0.5627	-0.8797 – -0.2457	**0.001**
Time	-0.0007	-0.0023 – 0.0009	0.404
Emotion x Time	0.0010	-0.0012 – 0.0032	0.383

Random Effects: Marginal R^2 = 0.004; Conditional R^2 = 0.038

N-1: Neutral

		Amplitude	
Predictors	***Estimates***	***CI***	***p***
(Intercept)	1.2988	0.8345 – 1.7631	**<0.001**
Emotion	-1.0220	-1.3511 – -0.6929	**<0.001**
Time	-0.0034	-0.0051 – -0.0017	**<0.001**
Emotion x Time	0.0019	-0.0004 – 0.0042	0.106

Random Effects: Marginal R^2 = 0.015; Conditional R^2 = 0.066

N-1: Mutilation

		Amplitude	
Predictors	***Estimates***	***CI***	***p***
(Intercept)	1.0115	0.5857 – 1.4372	**<0.001**
Emotion	-0.8233	-1.1407 – -0.5060	**<0.001**
Time	-0.0011	-0.0027 – 0.0005	0.167
Emotion x Time	0.0001	-0.0021 – 0.0023	0.929

Random Effects: Marginal R^2 = 0.014; Conditional R^2 = 0.059

Late Positive Potential: Mutilation vs. Neutral

N-1: Erotic

	Amplitude		
Predictors	***Estimates***	***CI***	***p***
(Intercept)	1.1295	0.6629 – 1.5962	**<0.001**
Emotion	-0.8546	-1.2431 – -0.4662	**<0.001**
Time	-0.0007	-0.0027 – 0.0012	0.453
Emotion x Time	0.0020	-0.0008 – 0.0047	0.157

Random Effects: Marginal R^2 = 0.006; Conditional R^2 = 0.039

N-1: Neutral

	Amplitude		
Predictors	***Estimates***	***CI***	***p***
(Intercept)	1.7962	1.3391 – 2.2532	**<0.001**
Emotion	-1.2430	-1.6363 – -0.8496	**<0.001**
Time	-0.0025	-0.0045 – -0.0005	**0.013**
Emotion x Time	0.0014	-0.0013 – 0.0042	0.312

Random Effects: Marginal R^2 = 0.017; Conditional R^2 = 0.047

N-1: Mutilation

	Amplitude		
Predictors	***Estimates***	***CI***	***p***
(Intercept)	0.7647	0.3259 – 1.2034	**0.001**
Emotion	-0.3974	-0.7929 – -0.0019	**0.049**
Time	-0.0002	-0.0022 – 0.0017	0.811
Emotion x Time	0.0007	-0.0020 – 0.0035	0.598

Random Effects: Marginal R^2 = 0.001; Conditional R^2 = 0.028
